# Exploratory Investigation of Intestinal Structure and Function after Stroke in Mice

**DOI:** 10.1155/2021/1315797

**Published:** 2021-02-15

**Authors:** Diya Ye, Yuting Hu, Ning Zhu, Weizhong Gu, Gao Long, Enfu Tao, Marong Fang, Mizu Jiang

**Affiliations:** ^1^Department of Gastroenterology, Children's Hospital, Zhejiang University School of Medicine, National Clinical Research Center for Child Health, Hangzhou 310052, China; ^2^Hebei North University, Zhangjiakou 075000, China; ^3^Institute of Neuroscience, Zhejiang University School of Medicine, Hangzhou 310058, China

## Abstract

Stroke is the second leading cause of death worldwide. Patients who have a stroke are susceptible to many gastrointestinal (GI) complications, such as dysphagia, GI bleeding, and fecal incontinence. However, there are few studies focusing on the GI tract after stroke. The current study is to investigate the changes of intestinal structure and function in mice after ischemic stroke. Ischemic stroke was made as a disease model in mice, in which brain and ileal tissues were collected for experiments on the 1^st^ and 7^th^ day after stroke. Intestinal motility of mice was inhibited, and intestinal permeability was increased after stroke. Hematoxylin-eosin (HE) staining showed the accumulation of leucocytes in the intestinal mucosa. Myeloperoxidase (MPO) activity and inflammatory proteins (nuclear factor kappa-B (NF-*κ*B), inducible nitric oxide synthase (iNOS)) in the small intestine were significantly increased in mice after stroke. The expression of tight junction (TJ) proteins (zonula occludens-1 (ZO-1), occludin, and claudin-1) was downregulated, and transmission electron microscopy (TEM) showed broken TJ of the intestinal mucosa after stroke. Glial fibrillary acidic protein (GFAP) and the apoptosis-associated proteins (tumor necrosis factor (TNF-*α*), caspase-3, and cleaved caspase-3) were notably upregulated as well. Ischemic stroke led to negative changes on intestinal structure and function. Inflammatory mediators and TNF-*α*-induced death receptor signaling pathways may be involved and disrupt the small intestinal barrier function. These results suggest that stroke patients should pay attention to GI protection.

## 1. Introduction

Stroke remains the second leading cause of death in the world and results in approximately 5.5 million deaths in the year 2016 [1, 2]. Stroke is divided into ischemic stroke and hemorrhagic stroke. In China, ischemic stroke accounts for 69.6%-77.8% of the total number of strokes [3]. Importantly, stroke may not only cause damage to the brain but also damage other organs such as the heart, lungs, and gastrointestinal (GI) tract [4]. The GI tract plays an important role in metabolic control and nutritional homeostasis [5]. Patients who have a stroke are susceptible to GI complications [4], such as dysphagia, GI bleeding, fecal incontinence, immunosuppression, and infections, which affect the outcome of stroke and increase the length of hospital stay and cost [1, 6]. The gut is a complex organ that is responsible for absorbing nutrients and it has its own immune and nervous system. The intestine lives in various stress events such as infections and trauma, and its damaged mucosa may increase the permeability which creates an environment suitable for spreading bacteria and endotoxins, which play an important role in developing systemic inflammatory response syndrome (SIRS) and multiple organ dysfunction syndrome (MODS) [7, 8]. More and more evidence confirms that there is a close relationship between the central nervous system, enteric nervous system, and GI tract [9]. The barrier function of the intestinal mucosa plays an important role in the brain-gut axis interaction. Hang et al. [10] reported the changes of intestinal mucosal structure in rats after traumatic brain injury, and Hotchkiss et al. [11] suggested that trauma and shock can cause apoptosis of intestinal cells and lead to impaired intestinal barrier function. Clinical or experimental evidences on intestinal function after stroke are limited and contradictory. For example, some researchers reported that stroke causes damage to the intestinal mucosa and increased intestinal permeability, resulting in translocation of the microbiota [12, 13], while others proposed that stroke does not alter the shape and function of the intestine [14]. In this study, we used an animal model to investigate the function and pathophysiology of the small intestine in the acute (1 day) and intermediate stages (7 days) of stroke and to explore its potential molecular mechanisms.

## 2. Materials and Methods

### 2.1. Animals

**Figure 1:**
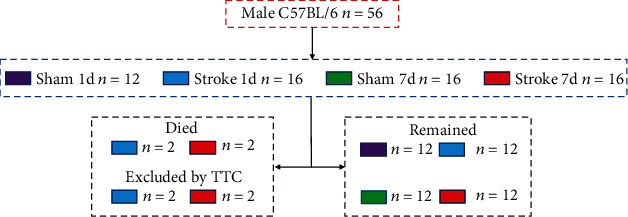
Animal grouping.

Male C57BL/6 mice of 8 weeks from SLAC (Shanghai, China) were used in this study. A total of 56 male mice were randomly divided into the following four groups: (i) sham 1-day group, (ii) stroke 1-day group, (iii) sham 7-day group, and (iv) stroke 7-day group (Figure 1). Before the experiment, the mice were housed in the experimental animal facility for one week for acclimatization. Mice lived in constant room temperature (24 ± 1°C), humidity (55%), 12-hour light-dark cycle, and specific pathogen-free (SPF) environment. During the entire experiment, sterilized food and water were made available unless otherwise stated. The experiments were carried out in accordance with the National Institutes of Health Guide for the Care and Use of Laboratory Animals. All experimental procedures were approved by the Zhejiang University Ethics Committee for Animal Research.

### 2.2. Surgical Procedures

Photochemically induced experimental stroke models were first developed by Watson et al. [15]. Unilateral cortical lesions were induced photochemically. Before the experiment, mice were anesthetized with 3% isoflurane/air (RWD Life Science, China) and maintained at 1.25% isoflurane during the surgery. The hair of the mice was shaved, and the head skin was sterilized with povidone. Then, the head skin was incised, and an optical fiber mounted on a cold light source (wavelength = 540 nm, *Φ* = 3 mm) was placed over the right hemisphere with a focus at 2 mm behind the bregma and 2 mm left to the midline. Rose Bengal (Sigma, America) was injected via the lateral tail vein, and then, we began focal illumination for 5 min. The wound was then sutured, and mice were returned to their cages. Animals in control groups underwent the same procedures, including light exposure and the surgery, but were administered with saline only. Finally, animals were euthanized 1 or 7 days later for brain and intestine (ileum) tissue collection.

### 2.3. TTC Staining and Neurological Deficit Scoring

Generally, 2,3,5-triphenyltetrazolium (TTC) reacts with normal brain tissue and is reduced to a red insoluble product. After euthanasia, the mouse brain was removed and sectioned into coronal slices (1 mm) and then immersed into 2% TTC solution at 37°C for 30 min. Stroke outcomes were evaluated with the Neurological Deficit Score (NDS) [12] which was a method of evaluating neurological deficit. The NDS was scored at ischemia, 1 and 7 days after surgery, using a 5-point scale: 0 point = normal; 1 point = when caught by the tail, the torso turns to the same side; 2 points = circle to the affected side; 3 points = difficult to support the weight of the affected side; and 4 points = no spontaneous movement.

### 2.4. Intestinal Propulsion Assay

Mice were gavaged with 0.2 ml of 10% methylene blue and anesthetized after 30 minutes. Laparotomy after euthanasia was performed, marking the end of the dye, and the impelling ratio (IR) was measured based on the length between the pylori and the end of the small intestine stained in blue (*B*) and the entire length of the small intestine (*L*). Intestinal propulsion was calculated according to the formula: IR = *B*/*L* × 100%.

### 2.5. Hematoxylin-Eosin (HE) Staining

Ileum segments were collected and put into 4% paraformaldehyde. Specimens were embedded in paraffin and cut into pieces. The 4 *μ*m thick sections were stained with HE staining and examined under a light microscope (Olympus BX61, Japan). Chiu's scoring system [16] was used to quantify the degree of intestinal damage following ischemic stroke: 0 point = normal mucosal villi; 1 point = development of the subepithelial space, usually at the apex of the villus; 2 points = extension of the subepithelial space with moderate lifting of the epithelial layer from the lamina propria; 3 points = massive epithelial lifting down the sides of villi; 4 points = denuded villi with lamina propria and dilated capillaries exposed; and 5 points = digestion and disintegration of lamina propria, hemorrhage, and ulceration.

### 2.6. Intestinal Permeability *In Vivo*

An intestinal permeability assay to assess the barrier function *in vivo* was performed using fluorescein isothiocyanate-dextran (FITC-D). Food and water were removed from cages for 4 hours, and animals then were gavaged with FITC-D solution (0.5 mg/1 g body weight, MW 4000; Sigma-Aldrich). One hour later, serum was collected. Fluorescence intensity of serum was measured and analyzed (excitation, 485 nm; emission, 525 nm) with a microplate reader (SynergyMx M5, Molecular Devices, America).

### 2.7. Quantitative Real-Time Reverse Transcription PCR

Total RNA was extracted from the tissue using the TRIzol reagent (Beyotime Biotechnology, China). Reverse transcription and quantitative PCR was carried out using the two-step Bestar qRCR RT Kit and CFX96 real-time PCR system (Bio-Rad, America). The PCR was conducted according to the manufacturer's protocol. Primer sequences are shown in Table 1. Relative expression level of the target gene was determined by the 2^−ΔΔCq^ method, and *β*-actin was utilized as an internal control.

### 2.8. Transmission Electron Microscopy (TEM)

The ileum for electron microscopy was fixed in 2.5% glutaraldehyde and 1% osmic acid. And then, the samples were stained with 2% uranyl acetate. Dehydration of the tissue was accomplished in ethanol at increasing concentrations. At last, specimens were embedded in an epoxy resin and made into specialized electron microscope sections. The ultrastructure of samples was examined under a transmission electron microscope (Tecnai G2 Spirit 120 kV, Thermo FEI).

### 2.9. Myeloperoxidase (MPO) Activity Assay

Myeloperoxidase activity was detected using the Myeloperoxidase Activity Assay Kit (Nanjing Jiancheng Bioengineering Institute, China). Experiments were performed according to the manufacturer's instructions. Briefly, ilea (50 mg weight) were isolated from each group and homogenized in 1 mL buffer solution. A reaction mix was prepared and added for each reaction according to the manufacturer's instructions. The samples were incubated at 37°C for 30 minutes. The reaction was stopped by adding 50 *μ*L stop mix to all samples and incubated at 60°C for 10 minutes. Light absorbance at 460 nm was read. MPO activity = (At − Ab)/(11.3 × *g*), where At is the absorbance value of the test group, Ab is the absorbance value of the blank group, and *g* is the weight of the sample.

### 2.10. Western Blot Analysis

Total protein of the intestinal tissues was extracted from each group using 0.5 mL ice-cold RIPA buffer, with added protease and phosphatase inhibitors (Rocher, Switzerland). After grinding twice with liquid nitrogen for 5 minutes each time and centrifuging at 12,000 rpm for 30 minutes at 4°C, the supernatant protein was collected and stored in a -80°C refrigerator. The protein concentration was determined using a BCA kit (Auragene Bioscience, China) and unified to 2.5 *μ*g/*μ*L. SDS-PAGE loading buffer was added to the protein sample (protein sample: loading buffer = 4 : 1), and boil the mixture in water at 100°C for 5 minutes. Then, a total of 20 *μ*g protein of each sample was subjected to electrophoresis on a 15% SDS-PAGE gel (Fude Biological Technology, China). After performing electrophoresis at 200 V for 60 min, the protein was electrophoretically transferred into a 0.45 mm polyvinylidene difluoride (PVDF) membrane by using a Bio-Rad TransBlot apparatus for 60 min at 300 mA. Afterwards, the PVDF membranes were blocked with TBST containing 5% skim milk for 3 hours at room temperature. Then, the membranes were incubated with different antibodies at 4°C overnight. The primary antibodies used were as follows: primary rabbit monoclonal against GAPDH (1 : 1,000; CST), ZO-1 (1 : 1,000; CST), occludin (1 : 1,000; CST), claudin-1 (1 : 1,000; CST), NF-*κ*B (1 : 1,000; CST), caspase-3 (1 : 1,000; CST), cleaved caspase-3 (1 : 1,000; CST), GFAP (1 : 1,000; CST), primary mouse polyclonal anti-TNF-*α* (1 : 400; Boster), and iNOS (1 : 400; Boster). After the incubation, the membrane was washed with TBST four times, each time for 5 minutes. The membrane was then incubated with goat anti-rabbit (1 : 5,000; BIOKER) and goat anti-mouse IgG antibodies (1 : 5,000; BIOKER) at room temperature (25°C) for 2.5 hours and then washed 4 times with TBST. Finally, the membranes incubated with enhanced chemiluminescence (ECL) were exposed in the ChemiDoc Touch Imaging System. The grayscale value of the protein band was analyzed by using Image Lab. All experiments were performed three times.

### 2.11. Immunofluorescence Staining

Frozen sections of 15 *μ*m thick tissue were dried at 37°C for 1 h. After blocking with 5% normal nonimmune goat serum for 1 h, sections were incubated at 4°C overnight with the following primary antibodies: GFAP (1 : 500, mouse IgG; CST). Then, sections were rinsed four times with PBS for 5 minutes each one and incubated with secondary antibodies (1 : 500, Dylight488 goat anti-rabbit IgG, EarthOx) for 2 h. Sections were then washed 3 times with PBS, and a mounting medium containing 4′,6-diamidino-2-phenylindole (DAPI) was added to the sections and then sealed with a coverslip. Fluorescent signals were observed under a fluorescence microscope (Olympus BX61, Japan). Three tissue sections were selected from each slide for analysis, and mean values were calculated. Independent experiments were repeated three times. Relative density = IOD/area.

### 2.12. Statistical Analysis

Statistical analysis was performed by using IBM SPSS 22.0 software. Data are expressed as mean ± standard deviation (SD). The Kruskal-Wallis ANOVA on ranks followed by Dunn's post hoc analysis, paired-sample *T*-test, and two-way ANOVA followed by Tukey's post hoc test were used to determine the statistical significance between multiple groups, when appropriate. *p* < 0.05 was accepted as statistically significant.

## 3. Results

### 3.1. Stroke Model Was Successfully Established, and Intestinal Motor Function of Mice Decreased after Stroke

**Figure 2:**
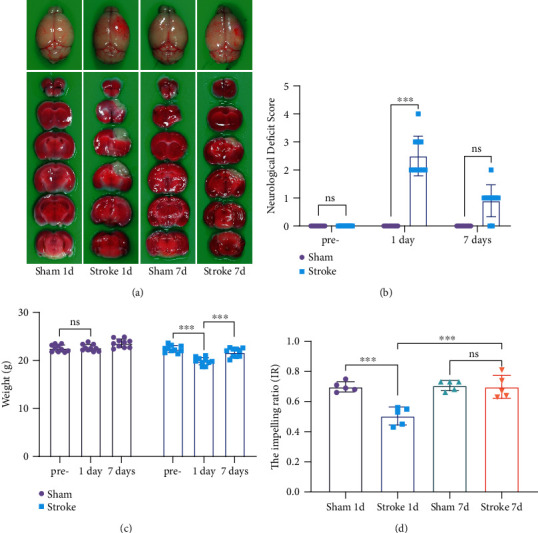
Ischemia model was successfully established. (a) Representative images of the mouse brain and TTC staining: the white part of the brain is the tissue of ischemic infarction. (b) Neurological Deficit Score (NDS) at different time points (*n* = 10) (Kruskal-Wallis ANOVA on ranks). (c) Mouse body weight at different time points (*n* = 10) (paired-sample *t*-test). (d) The impelling ratio (IR) at different times (*n* = 5) (two-way ANOVA with Tukey's multiple comparison test). Data were expressed as mean ± standard deviation. ^∗∗∗^*p* < 0.001. ns: not significant.

TTC staining showed an obvious cerebral infarction area in the experimental stroke model, including the stroke 1-day group and stroke 7-day group, while both 1-day and 7-day sham groups were completely normal (Figure 2(a)). The results showed that NDS was 0 point both before and after the surgery. NDS of the stroke group was significantly higher than that of the sham group (*n* = 10, *p* < 0.001, Figure 2(b)). One day after stroke, the weight of the mice decreased compared to prestroke (*n* = 10, *p* < 0.001, Figure 2(c)), but there was no difference in the sham group. The intestinal propulsion assay represented intestinal motor function, and the impelling ratio (IR) of the stroke 1-day group was lower than that of the sham 1-day group (*n* = 5, *p* < 0.001), while the stroke 7-day group was not statistically different from the sham 7-day group (*n* = 5, *p* > 0.05; Figure 2(d)).

### 3.2. Stroke Caused Intestinal Inflammation

**Figure 3:**
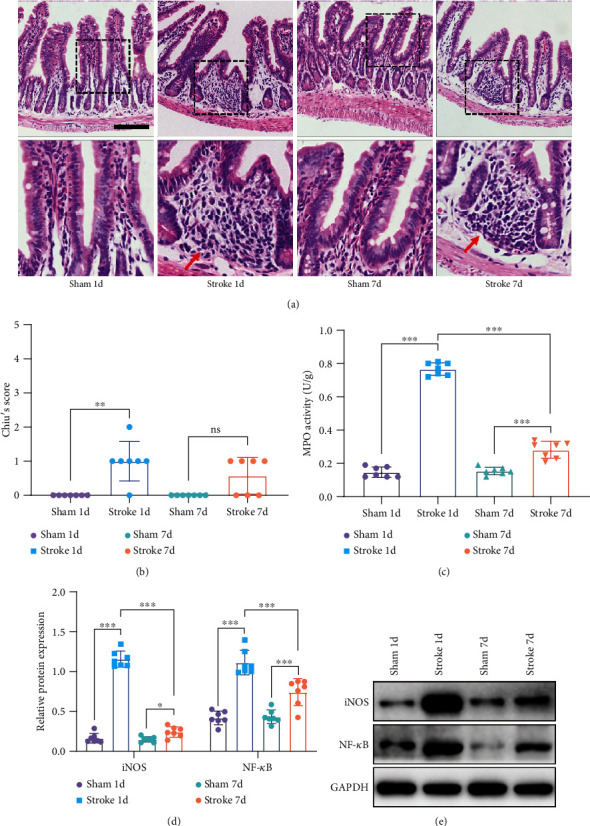
Stroke caused intestinal inflammation. (a) Representative images of hematoxylin-eosin (HE) staining of the ileum (magnification ×200, red arrow points to the leukocyte infiltration). (b) Chiu's score for the intestinal mucosa (*n* = 7) (Kruskal-Wallis ANOVA on ranks). (c) Myeloperoxidase (MPO) activity was measured (*n* = 7) (two-way ANOVA with Tukey's multiple comparison test). (d, e) Representative western blots and quantification data of iNOS, NF-*κ*B, and GAPDH for each group (*n* = 7) (two-way ANOVA with Tukey's multiple comparison test). Data were expressed as mean ± standard deviation. ^∗^*p* < 0.05, ^∗∗^*p* < 0.01, and ^∗∗∗^*p* < 0.001. ns: not significant. Scale bar: 100 *μ*m.

HE staining showed the accumulation of leucocytes in the intestinal mucosa (Figure 3(a), red arrow), but the intestinal villi were still intact. Chiu's score is recommended for pathological grading of intestinal mucosal villi. The results showed that the score of the stroke 1-day group was higher than that of the sham group (*n* = 7, *p* < 0.001, Figure 3(b)), and the scores were mainly on 1 point. The expression of MPO of the stroke 1-day group was higher than that of the sham 1-day group (*n* = 7, *p* < 0.001), and the level in the stroke 7-day group was higher than that in the control group while lower than that in the stroke 1-day group (*n* = 7, *p* < 0.01; Figure 3(c)). Both iNOS and NF-*κ*B expressions showed stronger bands in the stroke 1-day group than in the sham 1-day group (*n* = 7, *p* < 0.001; Figures 3(d) and 3(e)), and the levels in the stroke 7-day group were still higher than those in the sham 7-day group, but lower than those in the stroke 1-day group (*n* = 7, *p* < 0.001; Figures 3(d) and 3(e)).

### 3.3. Stroke Impairs the Function of the Intestinal Barrier Causing Increased Intestinal Permeability

**Figure 4:**
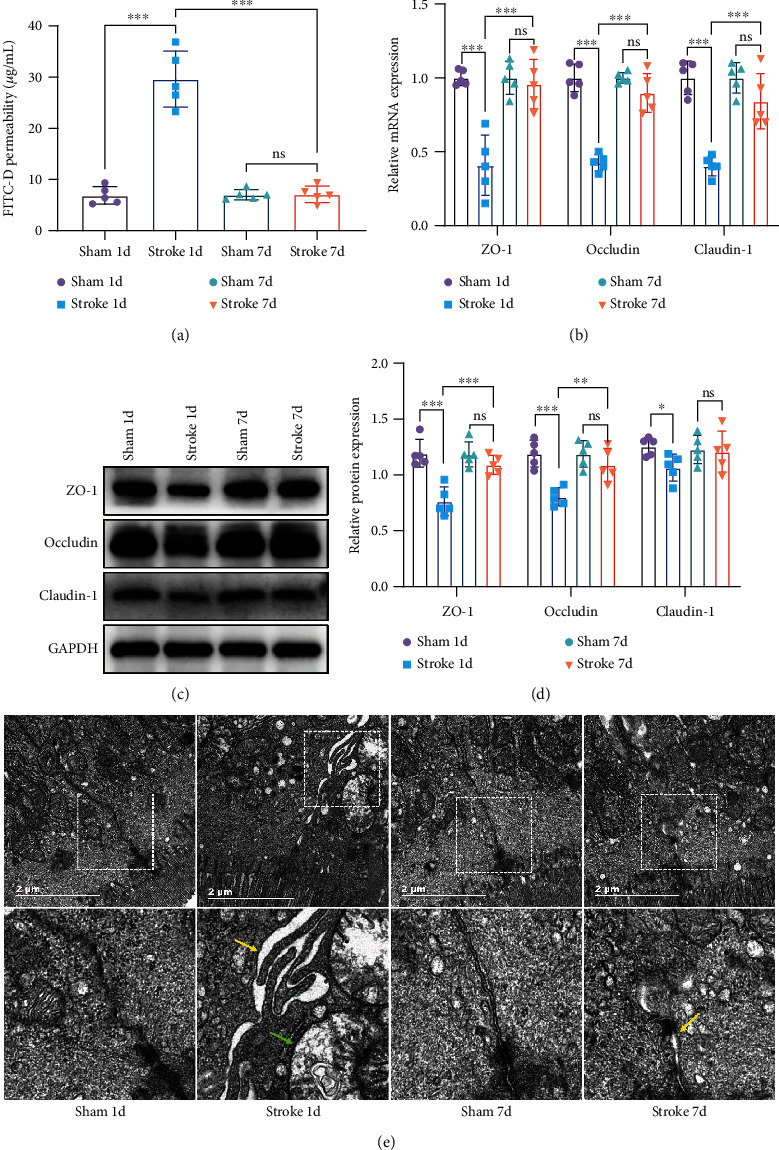
Ischemic stroke leads to increased intestine permeability. (a) Quantitative analysis of serum FITC-D as a measure of intestinal barrier functions (*n* = 5). (b) mRNA levels of ZO-1, occludin, and claudin-1 were measured with RT-qPCR and normalized to the expression levels of *β*-actin (*n* = 5). The results were presented as fold changes relative to the sham group. (c, d) Representative western blots and quantification data of ZO-1, occludin, claudin-1, and GAPDH for each group (*n* = 5). Two-way ANOVA with Tukey's multiple comparison test. Data were expressed as mean ± standard deviation. ^∗^*p* < 0.05, ^∗∗^*p* < 0.01, and ^∗∗∗^*p* < 0.001. ns: not significant. (e) Representative images of transmission electron microscopy (TEM) (magnification ×23000). The ultrastructure of the normal small intestinal mucosa looked like that of sham 1d and sham 7d, including perfect tight junctions (TJ) and normal mitochondria (yellow arrow points to enlarged tight junction, and green arrow points to degenerate mitochondria). Scale bar: 2 *μ*m.

The intestinal permeability of mice was measured by the FITC-D method. Compared with the sham 1-day group, the intestinal permeability of the stroke 1-day group was increased (*n* = 5, *p* < 0.001), while there was no significant difference between the stroke 7-day group and sham 7-day group (*n* = 5, *p* > 0.05; Figure 4(a)). The mRNA expressions of TJ proteins (ZO-1, occludin, and claudin-1) in mice decreased significantly in the stroke 1-day group (*n* = 5, *p* < 0.001, Figure 4(b)), but the difference had not reached significance after 7 days of stroke (*n* = 5, *p* > 0.05; Figure 4(b)). Western blot confirmed the qRT-PCR results (Figures 4(c) and 4(d)). Transmission electron microscopy (TEM) showed damage to TJ of the intestinal mucosa after stroke. Compared with the sham 1-day group, the space of TJ between epithelial cells in the stroke 1-day group was significantly enlarged (Figure 4(e), yellow arrow), and the internal structure of the cell was disordered, and its mitochondria appeared to have vacuole-like degeneration (Figure 4(e), green arrow). The space of tight junction in the stroke 7-day group had also expanded, but it was better than that in the stroke 1-day group (Figure 4(e)).

### 3.4. Stroke Activated Enteric Glia

**Figure 5:**
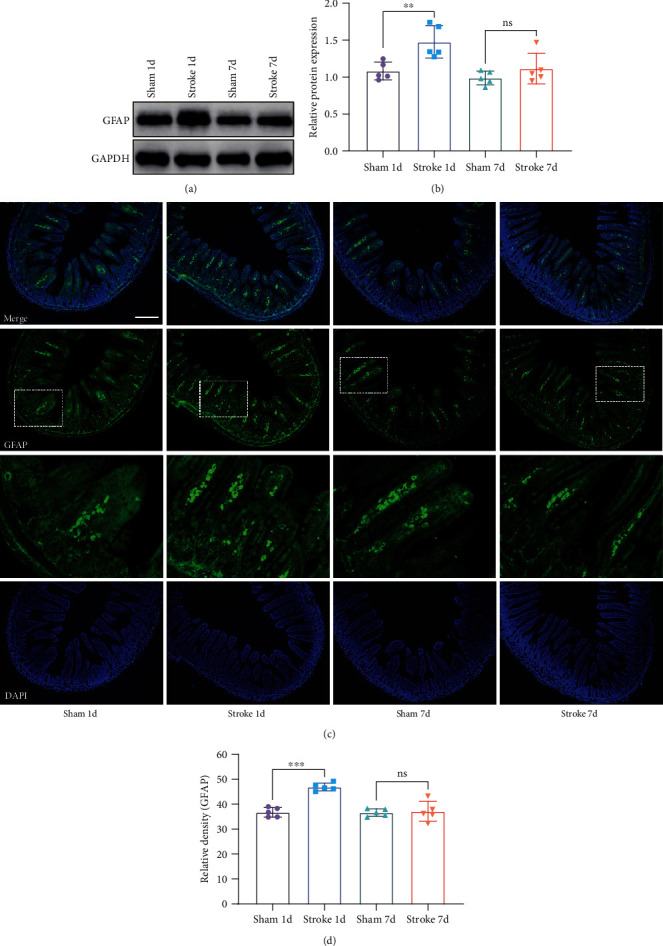
Ischemic stroke activated enteric glia. (a, b) Representative western blots and quantification data of GFAP and GAPDH for each group (*n* = 5). (c) Representative fluorescence microscopic images of GFAP (green) and DAPI (blue) (magnification ×100). Both images are merged at the top panel. (d) Quantification of GFAP punctae (*n* = 5). Data were expressed as mean ± standard deviation. ^∗∗^*p* < 0.01, ^∗∗∗^*p* < 0.001 (two-way ANOVA, Tukey's posttest). ns: not significant. Scale bar: 200 *μ*m.

Glial fibrillary acidic protein (GFAP) is a marker of astrocyte activation in the nervous system, which is also expressed in the intestine. GFAP protein expression in the stroke 1-day group was higher than that in the sham 1-day group (*n* = 5, *p* < 0.01; Figures 5(a) and 5(b)). However, there was no significant difference in GFAP expression between stroke 7-day and sham 7-day mice (*n* = 5, *p* > 0.05; Figures 5(a) and 5(b)). Immunofluorescence confirmed the western blot results (*n* = 5; Figures 5(c) and 5(d)).

### 3.5. Stroke May Promote Intestinal Apoptosis through the Death Receptor Signaling Pathway

**Figure 6:**
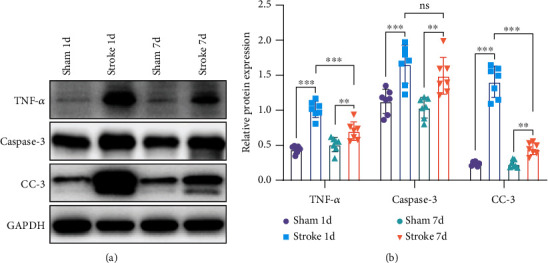
Ischemic stroke promotes apoptosis of the small intestine. (a, b) Representative western blots and quantification data of TNF-*α*, caspase-3, cleaved caspase-3, and GAPDH for each group (*n* = 7). Data were expressed as mean ± standard deviation. ^∗∗^*p* < 0.01, ^∗∗∗^*p* < 0.001 (two-way ANOVA with Tukey's multiple comparison test). ns: not significant; CC-3: cleaved caspase-3.

The protein expressions of TNF-*α*, caspase-3, and cleaved caspase-3 in the stroke 1-day group were stronger than those in the sham 1-day group (*n* = 7, *p* < 0.001; Figures 6(a) and 6(b)). The protein expression of the above three proteins in the stroke 7-day group was still stronger than that in the sham 7-day group but weaker than that in the stroke 1-day group (*n* = 7, *p* < 0.01; Figures 6(a) and 6(b)).

## 4. Discussion

The photochemically induced stroke model was first reported by Waston et al. in 1985 and continues to evolve [15, 17]. Rose Bengal releases active oxygen under light to damage the endothelium of blood vessels, which in turn causes platelets to aggregate and form thrombi. This model is stable and less invasive and is more in line with the pathophysiology of thrombosis [18, 19]. TTC staining results (Figure 2(a)) showed that the brains of the stroke group mice have large infarcted areas. The NDS is a method of evaluating neurological deficit, in which a score of 0 point is normal. It was shown that the mice developed neurological symptoms after stroke (Figure 2(b)). The weight of the mouse may be related to the damage suffered by the mouse. Intestinal propulsion experiments showed that stroke can significantly affect intestinal motility. Cerebral ischemia may cause an interruption of the axis between the GI system and central nervous system, leading to dysphagia and GI dysmotility [20]. Compared with the stroke 1-day group, the indicators of the stroke 7-day group were closer to those of the sham group (Figure 2(d)), which implies that motor functions of the mice are in a continuous recovery process after stroke. Intestinal motility is very complex and is affected in many ways, including nerves, hormones, inflammation, and bacteria [21–23]. Future studies are required in order to better explore these important issues.

Xu et al. [8] and Liu et al. [24] proposed that ischemic stroke may cause shedding and necrosis of the intestinal villus epithelium, while Oyama et al. [14] believe that the stroke will not cause changes in the small intestine pathophysiology and the appearance of these changes may depend on sectioning and the Swiss roll technique. The results of HE staining experiment in this study showed an accumulation of leucocytes in the intestinal mucosa (Figure 3(a), red arrow). Chiu's score showed minor damage to the small intestinal villi after stroke, while intestinal villi were still intact. In order to further study the internal mechanism of gut changes after stroke, we evaluated indicators related to mucosal inflammation and apoptosis. The experimental results showed that the protein expressions of inflammation-related proteins iNOS, NF-*κ*B, and MPO in the ileum were increased after 1 day of stroke (Figures 3(c)–3(e)). iNOS activated by bacterial pathogens or immunostimulating cytokines produces large amounts of NO via activation of the NF-*κ*B pathway, which is considered cytotoxic [25]. In the past decade, it has become increasingly agreed that upregulation of iNOS is harmful to the intestinal mucosa and iNOS plays an important role in the pathophysiology of DSS-induced colitis in mice [25–27]. The upregulation of iNOS and MPO reflects the increase in intestinal oxidative stress after stroke. The final effector of the TLR4 signaling pathway, NF-*κ*B, which plays a crucial role in the translation and transcription of inflammatory mediators, promotes the development of inflammatory bowel disease (IBD), and the TLR4/NF-*κ*B signaling pathway may be critical to the intestinal barrier function and mucosal inflammation [28, 29]. Neutrophil-MPO can catalyze the production of ROS. Upregulated ROS production is related to mucosal inflammation and may lead to damage to intestinal barrier function in IBD patients [30, 31]. Overexpressions of inflammatory mediators like iNOS, NF-*κ*B, and MPO suggested that the stroke caused a severe inflammatory response in the intestine, which was also accompanied by the time when the intestinal permeability increased.

The inner layer of the small intestine is composed of intestinal epithelial cells containing brush borders, villi, crypts, and so on. The tight junctions formed by neighboring epithelial cells are the basic structure of the intestinal mucosa, which constitutes a physical barrier and acts as a defense against antigens, bacteria, and xenobiotics [32]. We further examined the permeability of the small intestine and TJ proteins. We found that stroke caused increased permeability of the small intestine and decreased expression of TJ proteins (Figures 4(a)–4(d)). These results implied that the intestinal tight junction damage is caused by stroke. TEM was then performed to observe the ultrastructure of ileal tissue, such as organelle and microvilli. TEM results showed that the space of TJ between cells in the stroke 1-day group was significantly expanded (Figures 4(e), red arrow), and mitochondria appeared to have vacuole-like degeneration (Figure 4(e), green arrow). The space of TJ in the stroke 7-day group was still abnormal compared with that in the sham 7-day group (Figures 4(c) and 4(d)), but it was much better than that in the stroke 1-day group. Intestinal permeability tests also showed similar trends. The GI tract harbours a large number of bacteria, which exceed the number of host cells. Under normal physiological conditions, bacteria are the source of nutrients and maintain the dynamic balance of metabolism, which play an important role in the development and control of host immunity [33]. However, many diseases in our body are related to microbial disorders. Translocation or shifts in the makeup of these microbes can have significant pathological consequences for the host [33, 34]. It is reported that in a mouse model of ischemic stroke, poststroke infection was only observed in normal mice but not in mice that were born and raised in germ-free facilities, which implied that translocation of bacteria may be the cause of poststroke infection [13]. Bacterial infection was highly prevalent in stroke patients, which was thought to be related to weakened intestinal barrier function and intestinal flora translocation [35]. The damage to the intestinal mucosal cells after stroke may last for a while, and the process is reversible to some extent. Our study provided more direct evidence of impaired intestinal barrier function after stroke.

Astrocytes are the most abundant glial cell type within the central nervous system (CNS). During the acute phase of an ischemic stroke, astrocytes perform multiple functions both detrimental and beneficial for the survival of neurons [36]. Enteric glial cells (EGCs) are evidenced to be rich in glial fibrillary acidic protein (GFAP), a protein believed to be specific to astrocytes of the CNS. Accumulated data suggest that EGCs represent the morphological and functional equivalent of CNS astrocytes within the enteric nervous system (ENS) and may share with astrocytes the ability to modulate and integrate the neuronal activities [37, 38]. However, most studies focus on the effect of the activation of astrocytes in the brain after a stroke, while ignoring the activation of astrocytes in the intestine. It has been reported that the loss of enteric glia leads to a serious inflammation of the intestine [39]. Necrotizing enterocolitis (NEC) and IBD also show pathological changes in enteric glia [40]. EGCs are not only actors of gut diseases but also important regulators of intestinal barrier homeostasis [41]. We found that the protein expression of GFAP increased one day after the stroke but decreased to normal levels after 7 days (Figure 5). We speculate that it is the activation of enteric glial cells that causes inflammation and damage to the intestine. Stroke injury may firstly induce the activation of enteric glial cells and then cause the intestinal inflammation and apoptosis, further leading to the intestinal dysfunction, such as tight junction damage, increased permeability, and weakened intestinal motility. Enteric glial cells may be involved in the development of inflammation and play an important role in the restoration of intestinal balance. TNF-*α* plays a crucial role in the pathogenesis of IBD and NEC, and it is also the initiation factor of the death receptor pathway [42, 43]. Caspase-3 is a critical executioner of apoptosis, as it is responsible for proteolytic cleavage of many key proteins. IBD usually triggers inflammation by compromising barrier integrity, accompanied by an increase in TNF-*α* and cleaved caspase-3 levels [44, 45]. We found that protein expressions of intestinal TNF-*α* and apoptosis-related proteins caspase-3 and cleaved caspase-3 significantly increased after stroke in mice. TNF-*α* may activate the apoptosis process of the intestinal mucosa through the death receptor signaling pathway and then destroy the intestinal barrier function. The mechanism of changes in the GI tract after stroke is complicated. Xu et al. [8] believe that higher level of serum ghrelin may cause damage to the intestinal mucosa, while some scholars believe that vagus nerve stimulation may help reduce intestinal inflammation [46]. However, there are relatively few related studies, and its exact mechanism is still unclear and needs to be further explored.

Our study has demonstrated negative changes on intestinal structure and function, including inflammation of the intestinal mucosa, impaired tight junctions, and activation of enteric glial cells. However, the damage was reversible to some extent. Stroke can cause damage to both CNS and ENS, and enteric glial cells are indispensable for intestinal homeostasis. Indeed, the exact mechanism of gastrointestinal changes caused by stroke has not yet been determined and requires further study. Our study may help researchers understand the brain-gut axis regulation and has potential implications for the treatment of GI complications after stroke.

## Figures and Tables

**Table 1:** Primers used for RT-qPCR.

Gene	Primer	Primer sequence (5′-3′)
ZO-1	Forward	CATCATTCGCCTTCATAC
	Reverse	GTGTCTACTGTCCGTGCT
Occludin	Forward	CTTTGGCTACGGAGGTGGCTAT
	Reverse	CTTTGGCTGCTCTTGGGTCTG
Claudin-1	Forward	GCTGGGTTTCATCCTGGCTTCT
	Reverse	CCTGAGCGGTCACGATGTTGTC
*β*-Actin	Forward	CGTGCGTGACATCAAAGAGAAG
	Reverse	CAAGAAGGAAGGCTGGAAAAGA

## Data Availability

The datasets generated for this study are available on request to the corresponding author.
